# Impact of different privacy conditions and incentives on survey response rate, participant representativeness, and disclosure of sensitive information: a randomized controlled trial

**DOI:** 10.1186/1471-2288-14-90

**Published:** 2014-07-16

**Authors:** Maureen Murdoch, Alisha Baines Simon, Melissa Anderson Polusny, Ann Kay Bangerter, Joseph Patrick Grill, Siamak Noorbaloochi, Melissa Ruth Partin

**Affiliations:** 1 Section of General Internal Medicine, Minneapolis VA Medical Center, Minneapolis, MN, USA; 2 Center for Chronic Disease Outcomes Research, Minneapolis VA Medical, One Veterans Drive, Minneapolis, MN 55417, USA; 3 Department of Internal Medicine, University of Minnesota School of Medicine, Minneapolis, MN, USA; 4 Departments of Psychiatry and Psychology, Minneapolis VA Medical Center, Minneapolis, MN, USA; 5 Department of Psychiatry, University of Minnesota School of Medicine, Minneapolis, MN, USA

**Keywords:** Randomized trial, Patient surveys, Participation bias, Non-response bias, Anonymity, Confidentiality

## Abstract

**Background:**

Anonymous survey methods appear to promote greater disclosure of sensitive or stigmatizing information compared to non-anonymous methods. Higher disclosure rates have traditionally been interpreted as being more accurate than lower rates. We examined the impact of 3 increasingly private mailed survey conditions—ranging from potentially identifiable to completely anonymous—on survey response and on respondents’ representativeness of the underlying sampling frame, completeness in answering sensitive survey items, and disclosure of sensitive information. We also examined the impact of 2 incentives ($10 versus $20) on these outcomes.

**Methods:**

A 3X2 factorial, randomized controlled trial of 324 representatively selected, male Gulf War I era veterans who had applied for United States Department of Veterans Affairs (VA) disability benefits. Men were asked about past sexual assault experiences, childhood abuse, combat, other traumas, mental health symptoms, and sexual orientation. We used a novel technique, the pre-merged questionnaire, to link anonymous responses to administrative data.

**Results:**

Response rates ranged from 56.0% to 63.3% across privacy conditions (*p* = 0.49) and from 52.8% to 68.1% across incentives (*p* = 0.007). Respondents’ characteristics differed by privacy and by incentive assignments, with completely anonymous respondents and $20 respondents appearing least different from their non-respondent counterparts. Survey completeness did not differ by privacy or by incentive. No clear pattern of disclosing sensitive information by privacy condition or by incentive emerged. For example, although all respondents came from the same sampling frame, estimates of sexual abuse ranged from 13.6% to 33.3% across privacy conditions, with the highest estimate coming from the intermediate privacy condition (*p* = 0.007).

**Conclusion:**

Greater privacy and larger incentives do not necessarily result in higher disclosure rates of sensitive information than lesser privacy and lower incentives. Furthermore, disclosure of sensitive or stigmatizing information under differing privacy conditions may have less to do with promoting or impeding participants’ “honesty” or “accuracy” than with selectively recruiting or attracting subpopulations that are higher or lower in such experiences. Pre-merged questionnaires bypassed many historical limitations of anonymous surveys and hold promise for exploring non-response issues in future research.

## Background

Surveys represent one of the most efficient and inexpensive research methods available to collect representative, high quality data from large numbers of research participants. They therefore frequently serve as the backbone used to define the scope and magnitude of many potential public health problems. In the United States, for example, large national surveys have been used to estimate what proved at the time to be surprisingly high levels of mental illness within the general population [1], physical violence within families [2], and sexual assault among women [3]. Even the United States Census, which serves as the basis of apportioning Congressional representatives and taxes to each state, is survey-based. Typically, survey data are either collected by interviewers using face-to-face or telephone communication with the participant or via the participant’s own self-report.

Regardless of the topic studied and how the information is collected, scientifically correct, survey-based prevalence estimates require that research participants be representative of the population from which they are drawn, that participants actually answer the questions that are asked of them, and that they answer those questions honestly. On average, research participants disclose sensitive and personal information, such as mental health symptoms, drug misuse, and history of sexual assault more frequently when responding to self-administered questionnaires than when taking part in face-to-face or telephone interviews [4-7]. Studies suggest that disclosure of sensitive information on self-administered questionnaires is enhanced yet more when participants respond anonymously instead of confidentially [5,8-11]. This implies that anonymous, self-administered surveys may be the optimal method for accurately cataloging information about certain public health problems, such as the prevalence of physical or sexual abuse or of mental health symptoms.

Although by no means proven, most survey researchers take the stance that methods that generate higher prevalence estimates for stigmatizing or sensitive information are probably more accurate than methods that generate lower estimates. This stance, however, rests upon a rather unlikely assumption that all people carry the same propensity to participate in survey research. Particularly when a survey topic is sensitive, survey respondents tend to differ substantially from non-respondents [12]. Therefore, three mechanisms might explain why anonymous surveys generate higher prevalence estimates of stigmatizing or sensitive information compared to non-anonymous surveys: 1) propensity to participate in research is in fact equal across all members of a sampling frame, and anonymous methods promote more honest self-disclosure among the participants with stigmatizing experiences; 2) sampling frame members with stigmatizing experiences are more reluctant than others to participate in surveys, but anonymous methods reduce this inherent reluctance (under selection is reduced); 3) anonymous methods disproportionately increase the propensity of people with stigmatizing experiences to participate in the survey relative to those without such experiences (over selection is induced). The first two mechanisms reduce bias; the last introduces bias. Without information about non-respondents’ characteristics relative to respondents’, however, one cannot determine which possibility is correct. Unfortunately, under typical anonymous conditions, such information is unavailable.

Anonymous surveys carry other drawbacks relative to confidential surveys. For example, unlike confidential survey methods, anonymous survey responses cannot be linked to administrative or other non-survey data, thus limiting anonymous data’s richness and utility. Also, unless creative methods are employed, researchers often cannot track or send follow-up mailings to non-respondents of anonymous surveys, thus obtaining inferior response rates (e.g., [13]). While low response rates do not necessarily correlate to poor data quality, risks for non-response bias do increase with lower response rates.

Two methods to bypass the tracking limitation in anonymous surveys have been described. In one, participants return a completed survey and a separately mailed postcard. Only the postcard contains a unique identifier, which is used to track respondents [14-16]. However, this method increases respondent burden, which can reduce response rates. Furthermore, participants may find it confusing and hence return only one item –e.g., the survey or the postcard, but not both. Receipt of equal numbers of postcards and surveys do not necessarily mean the same people returned both. Even when both are returned by the same person, the survey may be received considerably earlier than the postcard. The participant may therefore be subjected to additional mailings until the postcard is received, which may be annoying, and the researcher may incur unnecessary mailing expenses. Finally, unbeknownst to the researcher, some respondents may return more than one survey, leading to the overweighting of those individuals’ responses.

A second approach uses tracking envelopes, which simplifies respondent burden, circumvents the problem of postcards and surveys returning at different times, and avoids analyzing multiple responses from a single participant [17]. In this approach, the envelope contains a unique identifier, but not the survey. The two are returned together but separated immediately upon opening. Received surveys are then intermixed in some random fashion to avoid any possibility of linking them back to their original envelopes. If one participant returns more than one envelope-survey pair, all but the first is discarded. Until the envelope and survey are separated, however, the survey is not truly anonymous. Participants must rely on the researcher’s integrity to maintain anonymity, and they may be less willing to disclose sensitive information relative to the postcard tracking method, where privacy is absolute. Each approach has pros and cons, but the two’s effect on response rates, survey completeness, or disclosure of sensitive information have never been directly compared.

In the present paper we address these issues using a novel technique we developed, the pre-merged questionnaire, which allows comparisons between respondents and non-respondents even under anonymous survey conditions. The study involved a potentially sensitive, self-administered questionnaire asking about several traumatic experiences, including sexual assault during military service. The population of interest was male US Gulf War I era veterans with possible posttraumatic stress disorder (PTSD) who had previously applied for Department of Veterans Affairs (VA) disability benefits. We had reason to believe that sexual assault experiences were particularly high in this population [18]. However, we also feared that traditional rape myth beliefs [19], which may be especially strongly held by military service members socialized into a masculinized subculture, might either deter male sexual assault survivors’ participation in the research or impede their disclosing of such experiences.

Using 3 levels of increasing privacy tied to the tracking methods described above, we hypothesized that response rate and participant representativeness, the number of sensitive questions actually answered by participants, and the proportion of participants disclosing potentially sensitive information would increase in a dose-response manner from the lowest to highest privacy condition. Because higher incentives consistently improve survey response [20], we also tested the impact of two incentives, $10 versus $20, on survey response. We expected the response rate, number of sensitive questions answered, and proportion of participants disclosing sensitive information would be higher among those receiving the $20 incentive compared to the $10 incentive.

## Methods

### Population and setting

We used simple randomization without replacement to select 324 veterans for survey from the population of 46,824 men who applied for VA PTSD disability benefits prior to June 2007 and had served in the US Armed Forces between August 2, 1990 and July 31, 1991.

### Study design and assignment

**Figure 1:**
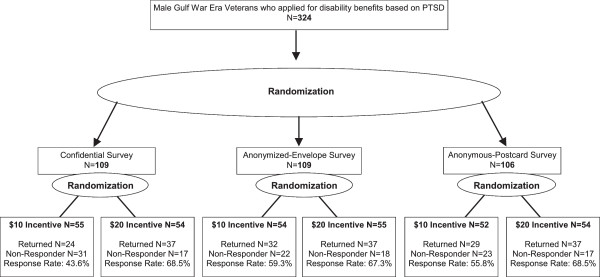
Study flow chart.

The study was a 3X2 factorial, randomized controlled trial (Figure 1). Using simple randomization, Veterans were assigned to one of 3 tracking/privacy conditions:

1) *“Confidential”*: Under the least private condition, veterans received a survey with a highly visible, coded, unique identifier affixed to the front page of the survey. This was used for tracking, and individual respondents were potentially identifiable from their surveys.

2) *“Anonymized-Envelope”*: Intermediate in privacy, veterans were asked to return their surveys in a study envelope, which had a pre-printed, unique identification number (ID) on it. When the completed questionnaire was returned, study personnel immediately separated it from the envelope. The questionnaire was intermixed with other arriving surveys and set aside. The envelope ID was used to indicate who had returned surveys. Technically, as long as the survey resided within the envelope, respondents could be identified. Thus, this method was not fully anonymous. Once the questionnaire was removed from the envelope, however, there was no longer any way to identify the respondent (hence the term “anonymized”).

3) *“Anonymous-Postcard”*: The most private condition, veterans returned their surveys in unmarked envelopes. Besides the survey, veterans were also asked to return an enclosed, brightly colored postcard, which had a unique ID to allow tracking. Respondents could not be identified from their surveys or envelopes at any time.

Once Veterans were assigned to their tracking/privacy condition, we then used simple randomization within each condition to assign them to receive $10 or $20.

### Protocol

#### Data collection

For all groups, the initial mailing included a cover letter describing the study’s risks and benefits, the cash incentive, and 25-page questionnaire. At two week intervals, non-respondents were mailed a post-card reminder, a second mailing of the survey, and a final mailing of the survey via overnight mail (Federal Express). Cover letters were printed on Minneapolis VA Medical Center letterhead and listed the study’s funding agency. Veterans were told that they had been selected for survey because they had filed a VA disability claim and had served during Gulf War I. They were also told that the survey would ask about “combat, unwanted sexual attention, and other lifetime and military experiences”. The cover letters also stated in bold-face font, “We would like to hear from you even if you never experienced combat or unwanted sexual attention. We would also like to hear from you even if you were not deployed to the Persian Gulf.” Cover letters were the same across groups, except that they described the incentive, tracking method, and privacy protections that were specific to each group. Copies of cover letters are available upon request.

#### Pre-merged questionnaires

**Figure 2:**
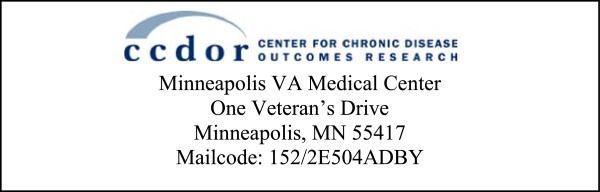
**Example of a pre-merged sticker.** For the present study, the sticker was placed within a pre-printed box on the last page of the survey. In this example, administrative data begins after the “E” in the Mailcode.

To our knowledge, we are the first to develop pre-merged questionnaires for use in anonymous surveys. However, pre-merged questionnaires are simply an extension of the common strategy of using different colored paper, say, to collect data from different groups (e.g., green paper for men, yellow for women). Instead of different colored papers, however, we created a sticker that was applied to each subject’s questionnaire just before mailing. The sticker was designed to be as unobtrusive as possible and was thus camouflaged as a return address on the survey’s back page (Figure 2). Just below the address, we embedded an alpha-numeric code into the mailcode, which corresponded to key administrative data associated with each potential subject. When the survey was returned, so was the administrative data—already merged. The sticker code was deliberately intended to be non-exclusive to the subject. For example, a code such as “504ADBY”, indicating a veteran was aged 50 years or older, served 4 years in the Army and received disability benefits from the VA, could apply to hundreds of thousands of veterans.

We maintained two separate, but interrelated computerized files: an administrative file containing subjects’ name and administrative codes, which were used to generate the stickers, and a tracking file containing their names and tracking ID. As envelopes, postcards, or confidential surveys were returned, the tracking ID was entered into the tracking file. This action deleted respondents’ name and ID from the tracking file and triggered a simultaneous deletion of their name and administrative code from the administrative file. Thus, by study’s end, only non-respondents’ administrative codes remained in the computerized record. These were then used to compare non-respondents to respondents. Respondents’ administrative codes were recaptured from the sticker on their returned questionnaires and hand-entered back into the analytical frame.

### Measures

#### Primary outcomes

The primary outcome was response rate, calculated as the number of returned surveys divided by the number of veterans assigned to each arm.

#### Secondary outcomes

Secondary outcomes included the representativeness of respondents, percentage of Veterans fully completing all sensitive survey items, and the percentage disclosing sensitive information. Information collected by the survey that we thought might be sensitive included veterans’ experiences of sexual abuse, including sexual assault during the time of Gulf War I; other traumatic experiences, including combat and childhood physical abuse; mental health problems, including depression, PTSD, and problem drinking; and veterans’ sexual orientation.

##### Representativeness of respondents

We used data from the pre-merged sticker to compare respondents to non-respondents. Available data included age greater than or equal to 50 years versus younger, service in the Army versus other branch, VA disability benefit status (receiving versus not), and any VA health care utilization versus none. Specifically, we assessed whether the participant had made a visit to any VA medical facility in the prior year for any reason or had made visits to a VA facility for primary or mental health care. The term, *original sample,* refers to all veterans selected for the survey, regardless of their response status. *Responders* and *respondents* refer to the subset of veterans from the original sample who returned surveys, and *non-responders/non-respondents* refer to the subset of veterans who did not return surveys.

##### Sensitive information

Sensitive information was collected by the survey and included the following:

Sexual abuse

We used 3 items from Sexual Harassment Inventory’s criminal sexual misconduct scale [21] plus one additional item [22] to assess sexual assault during the time of Gulf War I, 4 items from the Sexual Abuse subscale of the Childhood Trauma Questionnaire [23] to assess childhood sexual abuse, and one item from the Life Stressor Checklist [24] to assess any sexual assault in the past year. A positive response to any one of these questions indicated a history of sexual abuse.

Other traumatic experiences

Other traumatic experiences included combat exposure, assessed using an adapted Combat Exposure Inventory [25] version; childhood physical abuse, assessed using items from the Childhood Trauma Questionnaire’s relevant subscale [23]; and past-year traumas, assessed using an adaptation of the Life Stressor Checklist [24]. Veterans who reported any childhood physical abuse item more than “rarely” were considered physically abused.

Mental health problems

We used the 5-item RAND Mental Health Battery [26] to assess depression, the Penn Inventory for PTSD [27] to assess PTSD symptoms, and the TWEAK [28] to assess alcohol misuse.

Sexual orientation

Sexual orientation was assessed using a single survey item that read, “People are different in their sexual attraction to other people. Which best describes your feelings?” Responses ranged from *1* = “Completely heterosexual or ‘straight’” to *5* = “Completely homosexual or ‘gay’”. Responses were dichotomized as “Completely heterosexual” versus “Not completely heterosexual” for analysis.

### Power

The study was funded to examine different incentives’ impact on response rate and had 80% power to detect a 10% difference in response rates across incentives, assuming a 60% response rate in the $10 group and two-tailed alpha of 0.05.

### Analysis

The study was intended to examine main effects, but interactions were assessed in an exploratory fashion. Results are reported for tracking/privacy condition first; incentive condition second; and, when tested, interactions third. We used *χ*^2^ tests to compare outcomes across privacy conditions and incentives and to compare respondents and non-respondents. We used logistic regression to test for interactions between tracking/privacy condition and incentive on outcomes. We used IBM SPSS Statistics (version 19) and SAS (version 9.2) statistical packages for analyses.

### Masking, disclosure, and ethical approval

Data collectors and analysts were aware of study group assignment. The Minneapolis VA Medical Center’s Subcommittee for Human Studies approved the protocol.

## Results

### Response rate

Response rate overall was 60.5% and did not differ significantly across tracking/privacy assignments (Confidential response rate = 56.0%, Anonymized-envelope response rate = 63.3%, Anonymous postcard response rate = 62.3%, *p* = 0.49). However, the response rate was almost 15 full percentage points higher among veterans randomized to receive the $20 incentive (response rate = 68.1%) compared to the $10 incentive (response rate = 52.8%, *p* = 0.007). While the lowest response rate was obtained from men randomized to the Confidential/$10 incentive group (response rate = 43.6%; see Figure 1), tests for interactions between tracking/privacy and incentives on response rate were not statistically significant (*p* = 0.46).

#### Respondent representativeness

As Table 1 shows, randomization failed to evenly distribute the 324 veterans according to their past-year VA health care utilization. Specifically, veterans randomized to the Anonymous-Postcard were less likely to have made a VA health care visit of any kind in the past year than were veterans randomized to the Anonymized-Envelope and Confidential groups (67.6% versus 75.4% in the other two conditions). Otherwise, randomization successfully distributed all the remaining administrative characteristics evenly across all the tracking/privacy and incentive conditions.

**Table 1:** Population characteristics by tracking/privacy condition and incentive; results reported as a percentage (%)

**Characteristic**	**Overall**	**By tracking/privacy condition**			**Incentive**	
		**Confidential**	**Anonymized-Envelope**	**Anonymous-Postcard**	**$10**	**$20**
	***N*** **= 324**	***n*** **= 109**	***n*** **= 109**	***n*** **= 106**	***n*** **= 161**	***n*** **= 163**
Age > =50 yrs	35.8	32.1	42.2	33.0	40.4	31.3
Army service	63.0	67.0	59.6	62.3	65.8	60.1
Receiving VA disability benefits	83.3	83.5	83.5	83.0	86.3	80.4
VA visit last yr:						
Any type	74.1	76.1	80.7	65.1	77.0	71.2
Primary care	63.3	62.4	70.6	56.6	62.1	64.4
Mental health	47.5	50.5	54.1	37.7	51.6	43.6

The characteristics of survey responders are shown in Table 2. Responders in the Anonymized-Envelope group had a higher proportion of individuals aged 50 years or older, a lower proportion of white persons, and a lower proportion of persons working for pay compared to the other two groups, but none of these differences were statistically significant (all p’s > 0.18). Consistent with the original sample’s maldistribution, Anonymous-Postcard respondents were less likely than other respondents to have made a visit of any kind to a VA medical facility in the prior year. Compared to the other tracking/privacy conditions, Anonymous-Postcard respondents were also substantially less likely to have made a mental health care visit to a VA medical facility, but this could not be attributed to a maldistribution of the original sample. Compared to the administrative record, all respondents substantially under-reported receiving VA disability benefits.

**Table 2:** Respondent characteristics by tracking/privacy condition and incentive, results reported as a percentage (%)

**Characteristic**	**Overall**	**By tracking/privacy condition**			**By incentive**	
		**Confidential**	**Anonymized-Envelope**	**Anonymous-Postcard**	**$10**	**$20**
	***N*** **= 196**	***n*** **= 61**	***n*** **= 69**	***n*** **= 66**	***n*** **= 85**	***n*** **= 111**
Age > =50 years	44.4	42.6	50.7	39.4	56.5	35.1***
Race						
White	52.6	55.7	47.8	54.5	54.1	51.4
Black	27.0	21.3	34.8	24.2	22.4	30.6
Hispanic	6.1	8.2	5.8	4.5	8.2	4.5
Some college experience	74.5	75.4	73.9	74.2	76.5	73.0
Married	67.4	67.8	65.2	69.2	66.3	68.2
Working for pay	61.0	61.0	55.2	67.2	51.9	67.3*
Served in Army	60.2	63.9	56.5	60.6	63.5	57.7
Receiving VA disability benefits:						
Per the administrative record	84.2	90.2	82.6	80.3	87.1	82.0
Per self-report	68.4	75.4	71.0	59.1	76.5	62.2*
VA visit in past year:						
Any type	78.1	82.0	87.0	65.2*	81.2	75.7
Primary care	67.9	67.2	79.7	56.1	68.2	67.6
Mental health	51.0	54.1	65.2	33.3***	60.0	44.1

Bold face font signifies a statistically significant difference across group.

*p ≤ 0.05, ***p ≤ 0.001.

Respondents in the $10 incentive arm were significantly older, less likely to be working for pay, and more likely to say they received VA disability benefits than respondents in the $20 incentive arm. Both groups substantially underreported their receipt of VA disability benefits compared to the administrative record. There were no statistically significant tracking/privacy-by-incentive interactions (all *p*’s > 0.20).

Table 3 presents information for the original sample, stratified by response status and by study assignment. Findings show that Confidential and Anonymized-Envelope respondents differed significantly from their non-respondent counterparts in terms of age and service branch. Compared to their non-respondent counterparts, Confidential respondents were also more likely to be receiving VA disability benefits, and Anonymized-Envelope respondents were more likely to have made VA primary care and mental health visits. There were significant age differences among respondents and non-respondents randomized to receive $10, but respondents and non-respondents did not differ significantly on any available characteristic among those assigned to the Anonymous-Postcard or $20 incentive. There were no significant tracking/privacy-by-incentive interactions.

**Table 3:** Characteristics of original sample, stratified by response status and by tracking/privacy condition and incentive; results reported as a percentage (%)

**Characteristic**	**Overall**	**Overall by response**		**By tracking/privacy condition**						**By Incentive**			
				**Confidential**		**Anonymized-Envelope**		**Anonymous-Postcard**		**$10**		**$20**	
		***N*** **= 324**		***n*** **= 109**		***n*** **= 109**		***n*** **= 106**		***n*** **= 161**		***n*** **= 163**	
		**Respondent?**		**Respondent?**		**Respondent?**		**Respondent?**		**Respondent?**		**Respondent?**	
		**Yes**	**No**	**Yes**	**No**	**Yes**	**No**	**Yes**	**No**	**Yes**	**No**	**Yes**	**No**
	***N*** **= 324**	***n*** **= 196**	***n*** **= 128**	***n*** **= 61**	***n*** **= 48**	***n*** **= 69**	***n*** **= 40**	***n*** **= 66**	***n*** **= 40**	***n*** **= 85**	***n*** **= 76**	***n*** **= 111**	***n*** **= 52**
Age > =50 yrs	35.8	44.4	22.7***	42.6	18.8**	50.7	27.5*	39.4	22.5	56.5	22.4***	35.1	23.1
Army service	63.0	60.2	67.2*	63.9	70.8*	56.5	65.0*	60.6	65.0	63.5	68.4	57.7	65.4
Receiving VA disability													
benefits	83.3	84.2	82.0	90.2	75.0*	82.6	85.0*	80.3	87.5	87.1	85.5	82.0	76.9
VA visit last yr:													
Any type	74.1	78.1	68.0	82.0	68.8	87.0	70.0**	65.2	65.0	81.2	72.4	75.7	61.5
Primary care	63.3	67.9	56.2	67.2	56.2	79.7	55.0**	56.1	57.5	68.2	55.3	67.6	57.7
Mental health	47.5	51.0	42.2	54.1	45.8	65.2	35.0**	33.3	45.0	60.0	42.1	44.1	42.3

Bold face font signifies a statistically significant difference between respondents and non-respondents within that column.

*p ≤ 0.05, **p ≤ 0.01, ***p ≤ 0.001.

#### Percentage fully completing sensitive items and percentage disclosing sensitive information

As Table 4 shows, with the exception of combat items, respondents answered every item on each of the potentially sensitive scales more than 90% of the time, regardless of tracking/privacy condition or incentive. Twenty-six questions were used to assess combat exposure, which may explain why it had the most skipped items (10.7% overall), though respondents were twice as likely to skip a combat item as they were to skip a PTSD item (3.1% overall), which also contained 26 questions. The sexual abuse questions were second most likely to be skipped (7.1% overall). There were no statistically significant associations between tracking/privacy assignment and completion of sensitive survey items. Likewise, higher incentive was not associated with greater completion of sensitive survey items, and there were no interactions between tracking/privacy assignment and incentive.

**Table 4:** Percentage (%) of respondents fully completing all items in a potentially sensitive scale by tracking/privacy condition and incentive

**Scale/Item**	**Number of items in scale**	**Percentage (%) completing all items in the scale**					
		**Overall**	**By tracking/privacy condition**			**By incentive**	
			**Confidential**	**Anonymized-Envelope**	**Anonymous-Postcard**	**$10**	**$20**
		***N*** **= 196**	***n*** **= 61**	***n*** **= 69**	***n*** **= 66**	***n*** **= 85**	***n*** **= 111**
Sexual Orientation	1	98.5	100	98.6	95.5	97.6	98.2
Sexual Abuse	8	92.9	93.4	94.2	90.9	91.8	93.7
Other Traumatic events:							
Combat^*a*^	26	89.3	86.5	96.8	85.7	89.1	89.5
Childhood physical abuse	5	96.4	96.7	97.1	95.5	95.3	97.3
Past-year events:							
Economic hardship	1	98.5	100	97.1	98.5	97.6	99.1
Emotional abuse/neglect	1	99.0	98.4	100	98.5	98.8	99.1
Crime victim	1	99.0	100	98.6	98.5	97.6	100
Physical attack	1	98.5	98.4	98.6	98.5	97.6	99.1
Mental health screens							
Depression	5	99.0	100	98.6	98.5	98.8	99.1
PTSD	26	96.9	100	97.1	93.9	94.1	99.1
Problem drinking	5	100	100	100	100	100	100

^*a*^Among those who said they experienced any combat in the Gulf.

As Table 5 shows, Anonymized-Envelope respondents were substantially more likely than other respondents to disclose a history of sexual abuse. Several other contrasts appeared numerically large, even though they did not reach statistical significance: Anonymous-Postcard respondents reported more childhood physical abuse (*p* = 0.06) and had fewer positive depression screens compared to the other tracking/privacy groups (*p* = 0.09), and Confidential respondents reported more combat (*p* = 0.09) and had more positive PTSD screens (*p* = 0.08).

**Table 5:** Percentage (%) of respondents disclosing potentially sensitive information by tracking/privacy condition and incentive

**Sensitive information disclosed**	**Overall**	**By tracking/privacy condition**			**By incentive**	
		**Confidential**	**Anonymized-Envelope**	**Anonymous-Postcard**	**$10**	**$20**
	***N*** **= 196**	***n*** **= 61**	***n*** **= 69**	***n*** **= 66**	***n*** **= 85**	***n*** **= 111**
Not completely heterosexual	7.8	11.5	4.4	7.9	7.2	8.3
Any sexual abuse ever	20.9	14.8	33.3**	13.6	16.5	24.3
Other traumatic experiences						
Combat during Gulf War I	78.6	90.2	72.1	74.5	80.7	77.7
Childhood physical abuse	64.3	57.4	59.4	75.8	61.2	66.7
Past-year events:						
Economic hardship	43.3	41.0	42.6	46.2	39.8	45.9
Emotional abuse/neglect	22.6	23.3	25.7	18.5	26.2	19.8
Crime victim	10.8	9.8	13.0	9.2	9.6	11.6
Physical attack	5.2	6.7	7.2	1.5	1.2	0.9
Positive mental health screens:						
Depression	44.6	50.8	47.8	35.4	52.4	39.1
PTSD	79.2	85.2	77.1	75.8	83.5	75.9
Problem drinking	35.1	31.1	28.6	34.8	36.5	27.7

Bold face font signifies a statistically significant difference across groups.

**p ≤ 0.01.

Main effects in disclosing sensitive information by incentive did not reach statistical significance. However, there was a trend toward statistical significance in the proportion of respondents randomized to the $10 incentive with a positive depression screen compared to the $20 respondents (*p* = 0.08). Among Anonymized-Envelope respondents, those randomized to the $10 incentive were substantially more likely to screen positive for PTSD than those in the $20 arm (90.6% *v*. 65.8%; *p* = 0.05). Otherwise, there were no tracking/privacy-by-incentive interactions.

## Discussion

In this randomized controlled trial, more survey privacy was not associated with statistically significantly higher response rates compared to less privacy, nor did tracking/privacy condition affect the proportion of respondents who actually answered our sensitive questions. Instead, each tracking/privacy condition attracted its own unique pool of respondents, which in turn may have influenced our group-specific estimates of sexual abuse, childhood physical abuse, combat, and mental health problems—despite the fact that all participants originated from the same sampling frame. Estimates of sexual abuse, for example, were more than 2 times higher in the Anonymized-Envelope condition than in the other two conditions.

As expected, the higher incentive resulted in a substantially higher response rate than the lower incentive, but there was no association between incentive and the proportion answering our sensitive questions. As with the tracking/privacy manipulation, each incentive appeared to attract its own unique pool of respondents, with the larger incentive attracting younger workers for pay who were less likely to say they were receiving disability benefits compared to the smaller incentive. Statistically, prevalence estimates for potentially sensitive or stigmatizing material did not differ significantly by incentive, despite some numerically large differences. For example, more than half of respondents randomized to the $10 incentive screened positive for depression, compared to about a third of respondents in the $20 arm.

According to leverage-salience theory [29], individuals attend to different criteria when deciding to return a survey and, further, assign to each criterion different weights and importance. These are known as “leverages”. In the present study, each tracking/privacy and incentive condition appeared to trigger a different set of leverages, so that unique subpopulations selectively participated in each of the study’s arms. When considering sensitive material, therefore, one cannot assume that the survey method generating the highest estimate is most accurate.

Since Anonymous-Postcard respondents did not differ significantly from non-respondents on available measures, one might be tempted to conclude that this tracking/privacy method generated the most representative sample of respondents and hence most accurate prevalence estimates. If so, one would also have to conclude that the Anonymized-Envelope approach over recruited sexual abuse survivors. History of sexual abuse was 13.6% among Anonymous-Postcard respondents and 33.3% among Anonymized-Envelope respondents. However, we have shown elsewhere that, even when using Anonymized-Envelopes, survey respondents underreport their military sexual assault experiences by a factor of three [30]. This suggests that the Anonymized-Envelope method either reduces under selection of veterans with sexual abuse histories or optimizes more “honest” reporting among those who have such histories—or both—compared to the Anonymous-Postcard method. It may do so, however, at the expense of either over excluding veterans with a history of childhood physical abuse or discouraging “honest” reporting of childhood abuse. In the present study the Anonymized-Envelope method generated a substantially lower, albeit not statistically significant, estimate of childhood physical abuse of 59.4% compared to the Anonymous-Postcard’s estimate of 75.8%.

In general, tracking/privacy condition and incentive level appeared to affect respondent representativeness independently, with incentives’ principal impact being the recruitment of younger and healthier participants. These findings may be reassuring to Human Studies oversight boards, who might otherwise worry that large incentives coerce the sickest and most vulnerable into survey research participation. Halpern et al. [31] has shown that higher payment levels do not override research participants’ risk perceptions when considering whether to enroll in clinical trials, and, furthermore, poorer, presumably more vulnerable participants are actually less sensitive to higher incentive levels than are wealthier participants. Similar findings have been reported for those deciding whether to respond to a survey [32].

The present study offers proof-of-concept for pre-merged questionnaires’ utility. However, pre-merged questionnaires will prove most powerful when they incorporate administrative information that is highly related to the survey’s topic (e.g., sexual abuse, childhood abuse) instead of basic demographic information. Because we did not have such information for the present study, we cannot say whether our differing estimates for these sensitive data across the three tracking/privacy conditions were a function of reducing or inflating selection biases, a function of enhancing or impeding “honest” reporting, or both. Future research will be needed to explore these issues further. It may well be that different tracking/privacy methods will prove best for different sensitive topics.

We used a computerized system to manage the tracking and administrative data interface in the present study, but the pre-merged questionnaire concept could easily be applied to manual methods. For example in a study using up to three survey mailings per subject, one could pre-print 3 stickers per subject, file them under each subject’s name, and then throw away any remaining stickers once the subject’s postcard or envelope ID was returned. By study’s end, only non-respondents’ stickers would remain.

Pre-merged questionnaires carry important limitations. Researchers must be selective in what data they encode to keep the sticker from becoming uniquely identifying. If too much information is included, participants might become identifiable based on their unique combination of administrative data. We dichotomized age and service branch in the present study for this reason. Pre-merged questionnaires also cannot capitalize on new information. Health care visits occurring after a survey is mailed cannot be linked into a dataset, for example. Nonetheless, the technique offers an advance over usual anonymous methods, particularly in its ability to assess for non-response bias, and it could easily be applied to other sensitive topics.

This study’s strengths include its randomized, controlled design and demonstration of a unique technique to overcome what has historically been an important limitation of anonymous methods –namely, an inability to evaluate non-response bias. We also compared two tracking methods that can be used in anonymous surveys. Limitations include its relatively small and unique sample. Since we did not have access to verifying information, we cannot say how honestly participants reported their experiences. Findings’ generalizability to other sensitive topics, to non-veterans, or to women is also uncertain. The study was powered to examine main effects of incentives on response rates, and we may have made Type II errors when examining secondary outcomes, effects of the different tracking/privacy conditions, and potential interactions. When findings appeared suggestive, however, we described them in the text. We also made multiple comparisons, which may have inflated our Type I error.

## Conclusion

We anticipated that greater privacy and larger incentives would be associated with higher response rate, better participant representativeness, more survey completeness, and greater disclosure of potentially sensitive information. Results showed no association between privacy and response rate or survey completeness, supported the association between greater privacy and participant representativeness, and yielded mixed effects for the disclosure of sensitive information. A larger incentive was associated with higher response rate and better participant representativeness but no association with survey completeness. In the intermediate privacy arm, lower incentive—not higher—was associated with reporting more PTSD symptoms. Otherwise, we found no statistically significant associations between incentive and disclosing potentially sensitive information.

Having shown that different tracking/privacy conditions yielded different estimates of sensitive information, we cannot, unfortunately, tell which estimate was most accurate. Traditionally, higher disclosure rates of sensitive or stigmatizing information have been interpreted as being more accurate than lower rates, but our data suggest that apparently different disclosure rates may simply be a function of the subpopulations successfully recruited into a survey. This possibility needs greater investigation. Pre-merged questionnaires bypassed many of the limitations historically associated with anonymous survey methods and could be used to explore non-response issues in future research.

## Abbreviations

ID: Identification number; PTSD: Posttraumatic stress disorder; TWEAK: An acronym of 5 items used to assess problem drinking: T = tolerance, W = Worried, E = Eye-opener, A = Amnesia, K = Cut down; VA: Department of Veterans Affairs.

## Competing interests

The authors declare they have no competing interests.

## Authors’ contributions

MM obtained funding; designed the study; oversaw data collection, analysis, interpretation; and drafted the manuscript. MAP also assisted in obtaining funding. MAP, AKB, ABS, SN, and JPG contributed to data collection, analysis, and interpretation of data. MRP contributed to analysis and interpretation of data. MAP, AKB, ABS, SN, JPG, MRP read and approved the final manuscript.

## Authors’ information

MM, MAP, and MRP are core-investigators; AKB is data manager; and SN is core statistician for the Center for Chronic Disease Outcomes Research at the Minneapolis VA Medical Center. ABS is a former Center for Chronic Disease Outcomes Research data manager and currently works in the Health Economics Program, Minnesota Department of Health, St. Paul, MN. JPG is a former Center for Chronic Disease Outcomes Research statistician.

## Pre-publication history

The pre-publication history for this paper can be accessed here:

http://www.biomedcentral.com/1471-2288/14/90/prepub
